# Garba Dance Is Effective in Parkinson's Disease Patients: A Pilot Study

**DOI:** 10.1155/2024/5580653

**Published:** 2024-06-20

**Authors:** Anish Mehta, Pooja Dugani, Rohan Mahale, Nandakumar, Krishna Haskar Dhanyamraju, R. Pradeep, Mahendra Javali, Purushottam Acharya, R. Srinivasa

**Affiliations:** ^1^Department of Neurology, Ramaiah Medical College and Hospitals, Ramaiah University of Applied Sciences, Bengaluru, India; ^2^Medanta, Medicity, Gurgoan, India; ^3^Department of Neurology, National Institute of Mental Health and Neurosciences (NIMHANS), Bengaluru, India; ^4^Department of Community Medicine, Ramaiah Medical College, and Hospitals, Ramaiah University of Applied Sciences, Bengaluru, India; ^5^Medicover Hospital, Kakinada, India; ^6^Aster CMI Hospitals, Bengaluru, India

## Abstract

**Background:**

Dance as therapy is gaining attention as an adjuvant option for Parkinson's disease (PD). Assessing culturally popular dance forms is crucial for promoting the acceptance of dance therapy in a culturally sensitive context.

**Objectives:**

The pilot study evaluated the efficacy of Garba dance for alleviating motor symptoms, nonmotor symptoms, cognitive functions, and mood. Furthermore, this pilot study also assessed the tolerability and safety of this dance form using fatigue severity scale and assessing falls, respectively.

**Methods:**

Eligible participants with mild-to-moderate PD (H&Y stage 1 to 2.5) were sequentially randomized into the dance therapy, physical therapy, or control groups. Motor symptoms, nonmotor symptoms, cognitive functions, and mood were assessed using standard scales at baseline, week 6, and week 12. Tolerability was measured using the Fatigue Severity Scale, and safety was assessed by monitoring falls.

**Results:**

In the Garba dance group, significant improvements in UPDRS scores were observed at week 6 (*p*=0.002) and week 12 (*p* < 0.001) compared to baseline. At week 12, UPDRS scores were better in the Garba dance group as compared to the control and physical therapy groups. Freezing of gait significantly improved at week 6 (*p* < 0.001) and week 12 (*p* < 0.001) in the Garba dance group. Garba dance also led to significant improvements in mood and sleep. Tolerability was favorable, with significantly better fatigue severity scores in the Garba dance group at week 12 compared to controls and physical therapy. About 6 patients in Garba dance group experienced near falls.

**Conclusion:**

Underscoring a cautious optimism, results of the current study indicate that Garba dance may be an effective, safe, and well-tolerated intervention for Indian patients with mild-to-moderate PD (H&Y stage 1 to 2.5).

## 1. Introduction

Dopamine replacement therapy is the mainstay of treatment for Parkinson's disease [1]. However, the efficacy of these pharmacological therapies in controlling motor symptoms wears off with disease progression. Furthermore, appearance of nonmotor symptoms and their refractoriness to currently available pharmacological treatments are known to adversely impact health-related quality of life [2]. Overcoming these above limitations often necessitates a multidisciplinary approach comprising of surgical interventions, physical therapy, occupational therapy, cognitive therapy, and other rehabilitative interventions [3]. However, several barriers are known to impede adherence to these surgical and rehabilitative interventions, especially in older adults with Parkinson's disease [3]. Over the recent years, several complementary activities such as dance, music, theatre, art, and Tai Chi have been evaluated as therapeutic options for managing patients with Parkinson's disease [4–10]. Although evidence for the effectiveness of these therapies is currently nascent, initial results are promising, especially with respect to management of nonmotor symptoms, psychological well-being, and overall quality of life. In a recent systematic review, Sotomayer et al. point out that music therapy may have beneficial effects on motor symptoms, nonmotor symptoms, and cognition and emotional well-being [11]. Furthermore, studies by Mirabella et al. and Modugno et al. indicate that active theatre as a therapy may offer an effective form of holistic rehabilitation, wherein individuals with Parkinson's disease might learn or relearn social and emotional strategies in a protected environment and transfer them to everyday life situations [8, 9]. These studies also indicate that active theatre may also be associated with reduced depression, apathy, and stigma. Another study by Li et al. indicates that Tai Chi may offer beneficial effects on balance and frequency of falls [10]. Overall, these studies indicate that art forms employed as therapy may effectively complement standard treatment paradigms for Parkinson's disease.

Over the years, dance as a therapy has been gaining increasing attention as an option for people with Parkinson's disease [4, 5, 12–16]. When used as a therapy, dance is known to improve motor symptoms, nonmotor symptoms, cognitive functions, and increase striatal dopamine release [5]. Furthermore, dance comes across as an enjoyable physical activity, which can subvert the sedentary consequences for people with Parkinson's disease [4]. These features of dance as therapy may assist people with Parkinson's disease to shift their focus from impaired movements to the enjoyability of movements, which has important implications for compliance to treatments. Respectful inclusivity and sensitivities to diverse cultural backgrounds are important considerations when employing dance as therapy for people with Parkinson's disease. Several studies have reported that dance forms such as Irish set dancing, Argentine tango, Waltz/ Foxtrot are effective in improving balance, gait, and locomotion related aspects of PD [12–14]. Data on the usefulness of these dance forms for people with Parkinson's disease are more applicable for European, American, and Latin American settings. Applying dance as therapy in Indian settings require evaluation of Indian dance forms, and a few initial reports indicate that dance forms such as Bharatnatyam and Kathak may complement conventional treatments for Parkinson's disease [10, 11]. While these data are encouraging, it is important to note that Bharatnatyam and Kathak involve complex movements and extensive training, which may not be feasible in all patients with Parkinson's disease [15]. In Indian contexts, Garba dance, a popular dance form across India, which involves elegant footwork, hand gestures, and graceful movements of the body comes across as an easy-to-train and a relatively simple dance form.

On the above premise, the pilot study reported herein evaluated the efficacy of Garba dance for alleviating motor symptoms, nonmotor symptoms, cognitive functions, and mood. Furthermore, this pilot study also assessed tolerability of Garba dance using the fatigue severity scale, and safety of this dance form was assessed by monitoring falls.

## 2. Methods

This study was designed as an assessor-blind, randomized, three-arm, parallel group, single-center pilot study. As the nature of the study precluded double-blinding, patients and investigators were not blinded to treatment allocation. However, all assessments were made by raters who were blinded to treatment allocations at all time points in the study. This study was approved by the institutional ethics committee. People with idiopathic Parkinson's disease (stage 1 to stage 2.5 on Hoehn and Yahr staging system) aged between 30 and 80 years and willing to give an informed consent were included in the study. People with atypical Parkinson's disease; those with a known history of unstable cardiovascular status like arrhythmias/CCF, psychiatric conditions, osteoarthritis of knee, and respiratory Illness like asthma/COPD; those with a history of falls or head injuries in the last three months; and those with significant cognitive impairment (MOCA < 24) were excluded from the study. Following the procedures of informed consent, 55 eligible patients were sequentially allocated in a 1 : 1 : 1 manner to group A (Garba dance; *n* = 20), group B (physical therapy; *n* = 20), and group C (control; *n* = 15) using a computer-generated randomization sequence. Assessors were blinded to these allocations.

With respect to interventions, all patients were asked to continue their anti-Parkinsonian medications. Patients randomized to receive dance therapy (group A) were trained by a professional Garba dancer for a period of 1 week and subsequently underwent a few warmup sessions before study initiation. During these training sessions, patients were trained to perform Garba dance as part of group in a circular formation with rhythmic counterclockwise movements associated with sweeping actions of upper limbs. All these movements as part of Garba dance required patients to synchronize their movements to the tempo of associated music, which begins slowly with gradual increase in speed of movements. During the study period, patients performed Garba dance in 1-hour sessions for 5 days/week for a period of 12 weeks under supervision and assistance of trained professionals. All the dance sessions were conducted in a supervised environment at our center, and no dance sessions were done remotely or at home. To minimize the impact of falls, dance sessions during the warmup and experimental sessions were conducted on a specially prepared sponge-cushioned dance mat. Furthermore, dance assistants closely monitored the movements of all the patients in the dance group to avoid falls. Patients randomized to group B received 1-hour sessions of physical therapy for 5 days/week for a period of 12 weeks. Physical therapy was administered using 7 patterns of proprioceptive neuromuscular facilitation (PNF) technique to mimic Garba dance. Patients randomized to group C received standard pharmacotherapy. Efficacy and safety outcomes assessed in this pilot study included motor symptoms, nonmotor symptoms, balance, gait, activities of daily living (ADL), cognition, sleep, moods, fatigue, and falls. These assessments were performed at the baseline, week 6, and week 12. As this was a pilot study, no formal sample size calculation was performed. All assessments were performed by assessors blinded to the treatment groups.

Data were summarized, and proportions were compared using chi-square test, and means were compared using *t*-test or ANOVA. A repeated measures ANOVA, incorporating a within-factor analysis for time (3 levels) and a between-factor analysis for group (Garba dance, physical therapy, and control), was conducted. Tukey's post hoc tests were used to assess multiple comparisons across the groups. Cohen's d statistic was used to compute effect sizes for estimation of strength of treatment effects using an online tool available at https://www.statskingdom.com. The level of significance (*α*) was set at 0.05. All statistics were performed using IBM SPSS Statistics 22 software (IBM Corp 2013).

## 3. Results

The study included data from 55 patients (22 females and 33 males) with PD aged between 37 and 76 years. Although the original plan was to include 20 patients in each group to achieve an overall sample size of 60 for the entire study, we could recruit only 15 patients for the control group. The demographic and baseline characteristics of all patients are summarized in Table 1. There were no significant differences between groups at baseline with respect to demographic data, disease characteristics (stage, duration, and levodopa equivalent daily dose), and presence of comorbid conditions.

**Figure 1:**
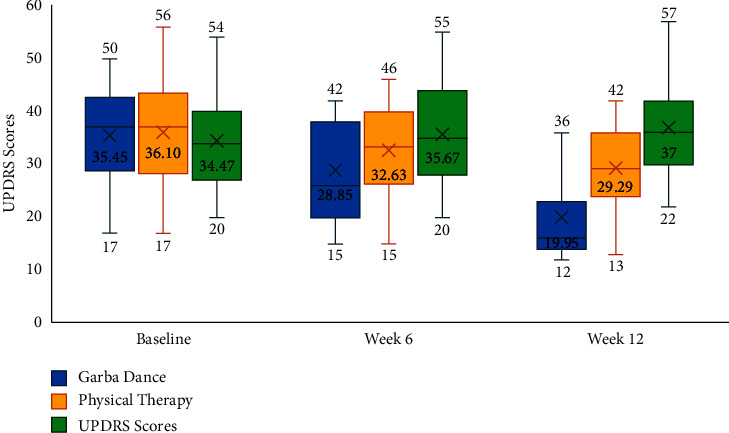
UPDRS at baseline, week 6, and week 12.

Results of repeated measures ANOVA indicated a statistically significant effect for UPDRS change over time (*η*^2^ = 0.296; *F* = 10.085; d*f* = 2, 48; *p* < 0.001) and the interaction effect between UPDRS change and the different treatment groups (*η*^2^ = 0.189; *F* = 5.70; d*f* = 4, 98; *p* < 0.001). Post hoc comparisons with Tukey tests suggested a significant difference in mean UPDRS scores between the control and Garba dance groups (mean difference = −7.628, *p* < 0.043), and no significant differences were found between the other group pairs.  Figure 1 and Table 2 present the within-group and between-group differences in UPDRS scores at baseline, week 6, and week 12, respectively.

**Figure 2:**
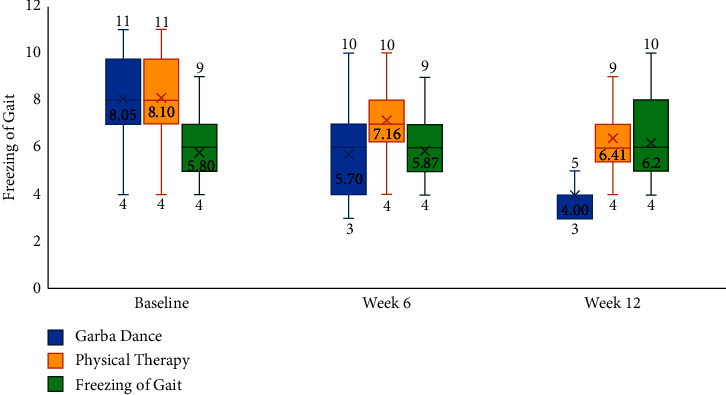
Freezing of gait at baseline, week 6, and week 12.

**Figure 3:**
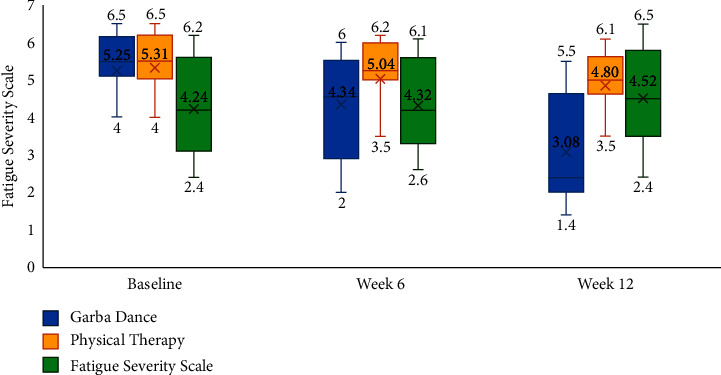
Fatigue severity at baseline, week 6, and week 12.

**Figure 4:**
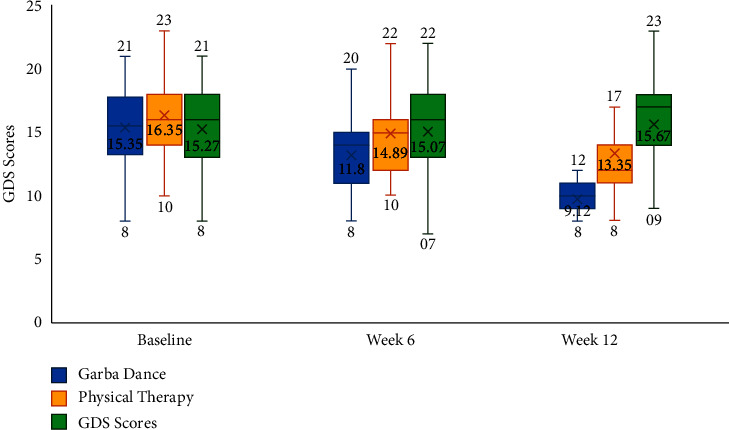
Mood as assessed by geriatric depression score at baseline, week 6, and week 12.

Furthermore, a statistically significant effect for change over time in freezing of gait (*η*^2^ = 0.417; *F* = 17.187; d*f* = 2, 48; *p* < 0.001), fatigue severity (*η*^2^ = 0.218; *F* = 6.54; d*f* = 2, 47; *p*=0.003), and mood as assessed by geriatric depression scale (*η*^2^ = 0.470; *F* = 21.269; d*f* = 2, 48; *p* < 0.001) was noted in the study. Furthermore, significant interaction effects were noted between the different treatment groups and freezing of gait (*η*^2^ = 0.417; *F* = 17.18; d*f* = 2, 48; *p* < 0.001), fatigue severity (*η*^2^ = 0.187; *F* = 5.536; d*f* = 4, 96; *p* < 0.001), and mood (*η*^2^ = 0.253; *F* = 8.301; d*f* = 4, 98; *p* < 0.001). With respect to post hoc comparisons, mood was significantly better in the Garba dance group as compared to physical therapy (mean difference = −3.42, *p* < 0.019) and control groups (mean difference = −4.05, *p*=0.006). Table 2 and Figures 2–4 present the within-group and between-group differences in freezing of gait, fatigue severity, and mood, respectively. While nonmotor symptoms, balance, activities of daily living, and cognitive functions showed minor improvements (Table 2), they were not statistically significant.

**Figure 5:**
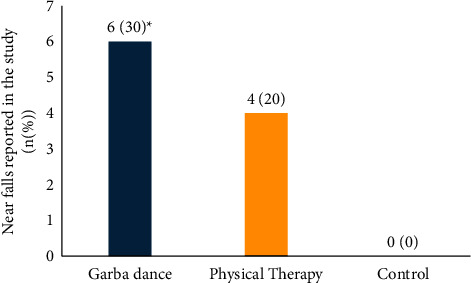
Incidence of near falls in the study.

Figure 5 depicts the incidence of near falls reported in the study. None of the falls were serious in nature and were not associated with any fracture or serious consequences. The dropout rates were 15% (3 out of 20) each in the Garba dance group and in the physical therapy group. The reasons for dropout included falls (1 out of 20 in the physical therapy group) and difficulties in travelling (2 out of 20 in the physical therapy group and 3 out of 20 in the Garba dance group).

## 4. Discussion

Results of the current study underscore a well-evidenced observation that a multidisciplinary approach involving pharmacotherapy, deep brain stimulation, and rehabilitative therapies is often needed for achieving outcomes in people with Parkinson's disease [1]. In this study, the mean change in UPDRS scores was better compared to baseline in the Garba dance group at week 6 and 12. However, mean change from in UPDRS scores in the physical therapy group and control groups was not significantly different at week 6 and week 12. Results of post hoc comparisons using the Tukey test noted that differences between groups were significant at week 12, wherein the people in the Garba dance group had better UPDRS scores as compared to physical therapy (*p*=0.031) and control group (*p* < 0.05). Furthermore, statistically significant improvements were noted for freezing of gait (Figure 2) and fatigue severity scores (Figure 3) at week 12. The percentage improvement in freezing of gait was 50.31 and 20.86 in Garba dance and physical therapy groups, respectively. Furthermore, percentage improvement in fatigue severity scores was 41.33 and 9.77 in Garba dance and physical therapy groups, respectively. These improvements are generally in line with the findings of another study by Hackney et al. which noted that Argentine tango and American ballroom dance both led to significant improvements in gait and balance and also observed an increase in dopamine release in the putamen following tango dancing [12].

The current study did not find any effect of employing Garba dance as therapy in improving nonmotor symptoms, activities of daily living (ADL), or cognition. However, results of the current study indicate that Garba dance may have a beneficial effect on mood (Figure 4) and sleep (Table 2). These findings are in agreement with other studies that have shown improvements in mood and reduction of depressive symptoms with dance therapy in individuals with Parkinson's disease [7, 14]. Volpe et al. note that the efficacy of dance as therapy may be attributable to the fact that it engages different brain regions such as the sensorimotor and limbic and cortico-basal ganglionic systems and promotes neural plasticity [14]. Furthermore, Hashimoto et al. in their article explain that dance as therapy may be more motivating and enjoyable than traditional exercise, leading to better adherence and long-term benefits [4]. Our observations showed that patients randomized to the Garba dance group did have an enjoyable time during the study. Thus, it appears that the enjoyability of Garba dance may lead to increased adherence to dance as therapy in people with an affinity for dancing and must therefore be seen as another option and not as a replacement for physical therapy in the overall multidisciplinary approach to managing patients with mild-to-moderate Parkinson's disease.

In this study, we used PNF techniques for administering physical therapy and intended it to mimic Garba dance movements, which involves limb movements in diagonal patterns. Furthermore, the PNF pattern of movements is diagonal movements that mimic functional activities of daily living as they involve movements characterized by crossing the midline of the body. Previous studies indicate that PNF techniques are effective in improving balance and gait [16]. In the current study, physical therapy was associated with significant improvements with respect to freezing of gait at week 12.

While there were no falls in the control group, 6 patients in the Garba dance group (*n* = 20) and 4 patients in the physical therapy group (*n* = 20) experienced near falls (Figure 5; *p*=0.072). Although the falls were completely avoided by having dance attendants closely monitor the patients during the dance and physical therapy sessions and none of the patients in this study experienced falls, it appears to be a concern and individuals with a susceptibility to falls must be excluded or must be monitored closely and sufficient precautions must be instituted. Cushioning the dance floor properly is a necessity for both clinical and research settings for dance therapies in PD. Nevertheless, in this study, the near falls were not associated with reasons for dropouts in the Garba dance group.

We specifically chose to include Garba dance over other Indian dance forms due to its relatively higher popularity and uncomplicated range of movements. Garba dance is a lively dance in a circular formation with counterclockwise movements associated with sweeping actions. The strong beats of the music for Garba stimulate engagement of body movement. Dancing begins slowly and increases in speed. The tempo of the music creates an energy that inspires rhythmic movement.

In this study, a notable decrease in their UPDRS scores (35.4 to 19.95 over a 12-week period) was noted in people allocated to the Garba dance. A key limitation of the current study is that lack of a premise to explain the magnitude of this UPDRS change. Data available from studies evaluating active theatre have indicated that individuals with Parkinson's disease might learn or relearn social and emotional strategies in a protected environment and transfer them to everyday life situations. Although data from the current study cannot provide a premise for such inferences, they does provide a suggestive premise for future studies to explore if such supervised environment to real-life transferences influences the change in UPDRS noted in the study [8, 9]. In this study, freezing of gait was seen in both the “off” and “on” states and more in the “off” state. However, this study did not capture the intergroup differences between the two states, and this may be another key limitation of this pilot trial. Furthermore, the operational constraints of the current pilot study did not allow for a post-study follow-up to check for the persistence of effects.

Overall, the results of this pilot study provide for a cautious optimism for considering Garba dance as an additional option in the multidisciplinary approach for managing people with mild-to-moderate Parkinson's disease. While the small sample size and the inherent limitations of a pilot study usher in caution, the noticeable improvements in UPDRS, freezing of gait, and fatigue severity scores along with improvements in mood and sleep lend premise for continued optimism. We computed Cohen's d to ascertain the effect size (change in UPDRS from baseline to week 12), which was −1.688. In a planned future study, we aim to detect an effect size of 0.8 with a power of 80% and a level of significance at 0.05 with allowances for 20% dropout. Thus, we plan to recruit 105 participants for the planned trial.

## Figures and Tables

**Table 1:** Baseline characteristics of patients included in the study.^*∗*^

	Group 1 (garba dance; *n* = 20)	Group 2 (physical therapy; *n* = 20)	Group 3 (controls; *n* = 15)	*p* value
Age (mean ± SD)	59.45 ± 9.37	59.35 ± 8.13	62.13 ± 8.21	0.581

Number of females (*n* (%))	8 (40)	8 (40)	6 (40)	1.000
Number of males (*n* (%)	12 (60)	12 (60)	9 (60)	

Stage 1^*∗∗*^ (*n* (%)	1	2	0	0.876
Stage 1.5^*∗∗*^ (*n* (%)	3	3	3	
Stage 2^*∗∗*^ (*n* (%)	7	7	7	
Stage 2.5^*∗∗*^ (*n* (%)	9	8	5	

LEDD (mean ± SD)^†^	619.80 ± 246.32	608.80 ± 283.59	599.20 ± 243.34	0.973

Duration of disease^††^ (mean ± SD)	39.15 ± 11.65	39.15 ± 11.65	36.80 ± 11.23	0.798

Smokers (*n* (%))	5 (25)	5 (25)	3 (20)	0.927

Alcoholism (*n* (%))	7 (35)	4 (20)	4 (26.66)	0.566

Diabetes (*n* (%))	7 (35)	7 (35)	5 (33.33)	0.993

Hypertension (*n* (%))	6 (30)	5 (25)	6 (40)	0.633

^*∗*^Means were compared using ANOVA and proportions were compared using chi-square test. ^*∗∗*^H&Y stage: Hoehn and Yahr staging system; ^†^LEDD: levodopa equivalent daily dose; ^††^duration of disease in months.

**Table 2:** Mean change in outcome measures assessed in the study.

Outcomes assessed	Scales used	Time points	Group 1 (garba dance; *n* = 20)	Group 2 (physical therapy; *n* = 20)	Group 3 (controls; *n* = 15)	*p* value
Motor symptoms	MDS-UPDRS	Baseline	35.45 ± 9.20	36.1 ± 9.66	34.47 ± 9.2	0.878
		Week 6	28.85 ± 12.66	32.63 ± 8.53	35.67 ± 9.81	0.172
		Week 12	19.95 ± 13.44	29.29 ± 8.3	37 ± 9.22	**<0.001**

Nonmotor symptoms	NMSS	Baseline	61.15 ± 27.02	56.20 ± 24.55	54.47 ± 20.69	0.697
		Week 6	53.80 ± 32.06	52.84 ± 24.34	55.07 ± 21.60	0.972
		Week 12	42.25 ± 32.38	49.29 ± 24.87	57.07 ± 21.83	0.291

Freezing of gait	Freezing of gait questionnaire	Baseline	8.05 ± 1.88	8.10 ± 1.77	5.80 ± 1.47	<0.05
		Week 6	5.70 ± 2.92	7.16 ± 1.64	5.87 ± 1.55	0.93
		Week 12	4.00 ± 2.75	6.41 ± 1.87	6.2 ± 1.82	0.003

Fatigue severity	Fatigue severity scale	Baseline	5.25 ± 1.14	5.31 ± 1.16	4.24 ± 1.34	0.022
		Week 6	4.34 ± 1.86	5.04 ± 1.19	4.32 ± 1.28	0.270
		Week 12	3.08 ± 2.12	4.80 ± 1.25	4.52 ± 1.25	0.006

Mini-BEST	Mini-BESTest balance evaluation	Baseline	18.25 ± 3.50	18.80 ± 3.35	20.07 ± 3.15	0.285
		Week 6	17.35 ± 6.83	19.11 ± 3.28	20.00 ± 3.02	0.264
		Week 12	16.25 ± 10.01	20.71 ± 3.46	20.00 ± 3.11	0.107

ADL	Schwab & England activities of daily living scale	Baseline	60.50 ± 12.34	60.50 ± 12.34	68.00 ± 8.61	0.107
		Week 6	60.00 ± 23.39	64.74 ± 11.23	67.33 ± 10.32	0.419
		Week 12	58.00 ± 31.55	70.59 ± 12.48	63.33 ± 11.75	0.227

Cognition	SCOPA-COG scale	Baseline	30.15 ± 4.52	31.15 ± 5.10	32.27 ± 4.38	0.425
		Week 6	27.40 ± 12.45	32.21 ± 5.17	31.93 ± 4.63	0.161
		Week 12	26.20 ± 15.85	33.25 ± 5.72	31.26 ± 5.10	0.124

Mood	Geriatric depression scale	Baseline	15.35 ± 3.49	16.35 ± 3.16	15.27 ± 3.88	0.573
		Week 6	11.20 ± 5.89	14.89 ± 3.46	15.07 ± 4.16	**0.023**
		Week 12	9.12 ± 5.03	13.35 ± 3.57	15.67 ± 4.11	**<0.001**

Sleep	Parkinson's disease sleep scale	Baseline	28.25 ± 6.31	28.40 ± 6.39	27.07 ± 5.92	0.800
		Week 6	22.05 ± 9.45	25.95 ± 6.02	27.87 ± 6.42	**0.074**
		Week 12	16.40 ± 11.10	24.29 ± 5.9	28.47 ± 6.44	**<0.001**

The bold values indicate statistically significant values.

## Data Availability

Readers can request the corresponding author for the data supporting the conclusions of the study.
